# The preoperative Hounsfield unit value at the position of the future screw insertion is a better predictor of screw loosening than other methods

**DOI:** 10.1007/s00330-022-09157-9

**Published:** 2022-10-14

**Authors:** Jingchi Li, Zhuang Zhang, Tianhang Xie, Zhetao Song, Yueming Song, Jiancheng Zeng

**Affiliations:** 1 Department of Orthopedic Surgery and Orthopedic Research Institute, West China Hospital/West China School of Medicine, Sichuan University, 37# Wuhou Guoxue Road, Chengdu, 610041 Sichuan Province People’s Republic of China; 2 Department of Imaging, Sichuan University, Chengdu, Sichuan People’s Republic of China

**Keywords:** X-ray computed tomography, Internal fixators, Bone density, Spinal fusion, Bone screws

## Abstract

**Objective:**

Screw loosening is a widely reported issue after spinal screw fixation and triggers several complications after lumbar interbody fusion. Osteoporosis is an essential risk factor for screw loosening. Hounsfield units (HU) value is a credible indicator during bone mineral density (BMD) evaluation. As compared with the general evaluation of BMD, we hypothesized that specific measurements of HU at the precise location of the future screw insertion may be a better predictor of screw loosening.

**Methods:**

Clinical data of 56 patients treated by oblique lumbar interbody fusion (OLIF) of the L4-L5 segments with an anterior lateral single rod (ALSR) screw fixation were reviewed in this study. Vertebral bodies with ≥ 1 mm width radiolucent zones around the screw were defined as screw loosening. HU in the insertional screw positions, the central transverse plane, and the average values of three and four planes were measured. Regression analyses identified independent risk factors for screw loosening separately. The area under the receiver operating characteristic curve (AUC) was computed to evaluate predictive performance.

**Results:**

The local HU values were significantly lower in the loosening group, regardless of the selected measuring methods. The AUC of screw loosening prediction was higher in the insertional screw positions’ HU than other frequently used methods.

**Conclusions:**

The HU value measured in the insertional screw position is a better predictor of ALSR screw loosening than other methods. The risk of screw loosening should be reduced by optimizing the trajectory of the screw based on the measurement of HU in preoperative CT.

**Key Points:**

*• Osteoporosis is an essential risk factor for screw loosening, and Hounsfield units (HU) are a credible predictor during bone mineral density (BMD) evaluation.*

*• The HU value measured in the insertional screw position is a better predictor of screw loosening than other frequently used HU measurement methods.*

*• The risk of screw loosening might potentially be reduced by optimizing the trajectory of the screw based on the measurement of HU in preoperative CT.*

## Introduction

Screw fixation is a standard surgical method in spinal surgery [1–3]. With the rapid promotion of oblique lumbar interbody fusion surgery (OLIF), different anterior-lateral single rod (ALSR) screw fixation devices have been promoted to reconstruct instant postoperative stability in a single incision [1, 4]. As a hardware-related complication, screw loosening is common, which may negatively affect the local mechanical environment, triggers several postoperative complications (e.g., surgical segmental instability, nonunion, and pseudarthrosis), and deteriorates the patient's long-term outcome [5–7]. Osteoporosis is common, especially for elderly patients, and is an essential risk factor for screw loosening [4–6]. Dual-energy X-ray absorptiometry (DXA) is the gold standard for its detection [8, 9]. However, new formation, like osteophytes, for instance, may influence the results of the T-score, leading to underestimation of cancellous bone damage, and therefore potential screw loosening [10–16].

Measurements of vertebral Hounsfield unit (HU) value on preoperative computed tomography (CT) is a credible indicator to evaluate spinal BMD and diagnose osteoporosis [6, 8, 9]. HU values are measured in the vertebral body, at the midsagittal plane, central transverse plane, and transverse planes close to the superior and inferior endplates separately [17, 18]. In this process, the region of interest (ROI) is expanded as much as possible within the cancellous bone but excluding other bony structures, such as cortical, bony endplates, and osteophytes. There are three standard methods: the value of the central transverse plane, the three planes’ average value (average value of three transverse planes), and the four planes’ average values (average value of both three transverse planes and the midsagittal plane) [3, 16, 17]. The confusion caused by pathological bone formations can be eliminated [8, 18, 19], and the specific BMD of cancellous bone can be measured [3, 16, 17]. Based on the advantages mentioned above, it is a better predictor of postoperative complications than the T-score [8–10] and is widely used in the prediction of screw loosening. Its predictive performance is better than that of DXA [8, 10, 16].

However, HU measurement methods still have inherent drawbacks when evaluating the risk of screw loosening. The damage of local cancellous microstructures triggers the loss of screw-bone integration and resulting in a higher risk of screw loosening [7, 15]. Considering that noticeable regional differences exist [20, 21], HU measurement in specific planes may mask the differences in different regions. Several studies reported that HU measured in the cancellous bone of pedicle screws has a better predictive performance of screw loosening than other methods [22–25]. Based on the above theoretical and practical foundations, we hypothesized that the HU value of the insertional screw position for ALSR is a better predictor of screw loosening than other HU measurement methods. However, this topic has not been verified in published studies. This study aimed to verify this hypothesis and provide theoretical guidance for the screw trajectory optimization of ALSR fixation.

## Materials and methods

### Patient data collection

Approval for the current study protocol was obtained from the ethics committees of our hospital (2020-554). Informed consent was waived for this retrospective study. We retrospectively reviewed demographic and radiographic data of 56 patients who underwent OLIF with ALSR screw fixation and without posterior decompression in the L4-L5 motion segment from May 2017 to August 2019. The average follow-up period of these patients was 12.2 months (within 11 to 13.5 months). The OLIF operation in these patients was performed by a senior spine surgeon. Screw types and sizes were completely identical. All screws were placed in a single attempt, and all screws penetrated the contralateral cortex.

The inclusion criteria were as follows: (1) Patients who underwent OLIF with ALSR screw fixation for lumbar degenerative diseases, including spinal stenosis and grade 1 and grade 2 degenerative spondylolisthesis; (2) Patients who underwent lumbar CT three times, including 1 week before, 1 week after, and approximately 1 year (within 11 to 13.5 months) after OLIF surgery. The exclusion criteria were as follows: (1) Patients with a history of lumbar surgery; (2) Patients with primary or metastatic spinal tumors, lumbar tuberculosis, rheumatic immune diseases, and secondary osteoporosis caused by medication or other metabolic diseases; (3) Patients with grade 3 and grade 4 degenerative spondylolisthesis or any grades of spondylolysis (i.e., isthmic spondylolisthesis); (4) Patients with additional posterior approach decompression; (5) Patients who underwent lumbar revision surgery within the clinical follow-up period of 12 months for complications other than screw loosening; (6) Patients who underwent intraoperative screw replacement.

### Assessment of screw loosening and HU measured by different methods

**Fig. 1:**
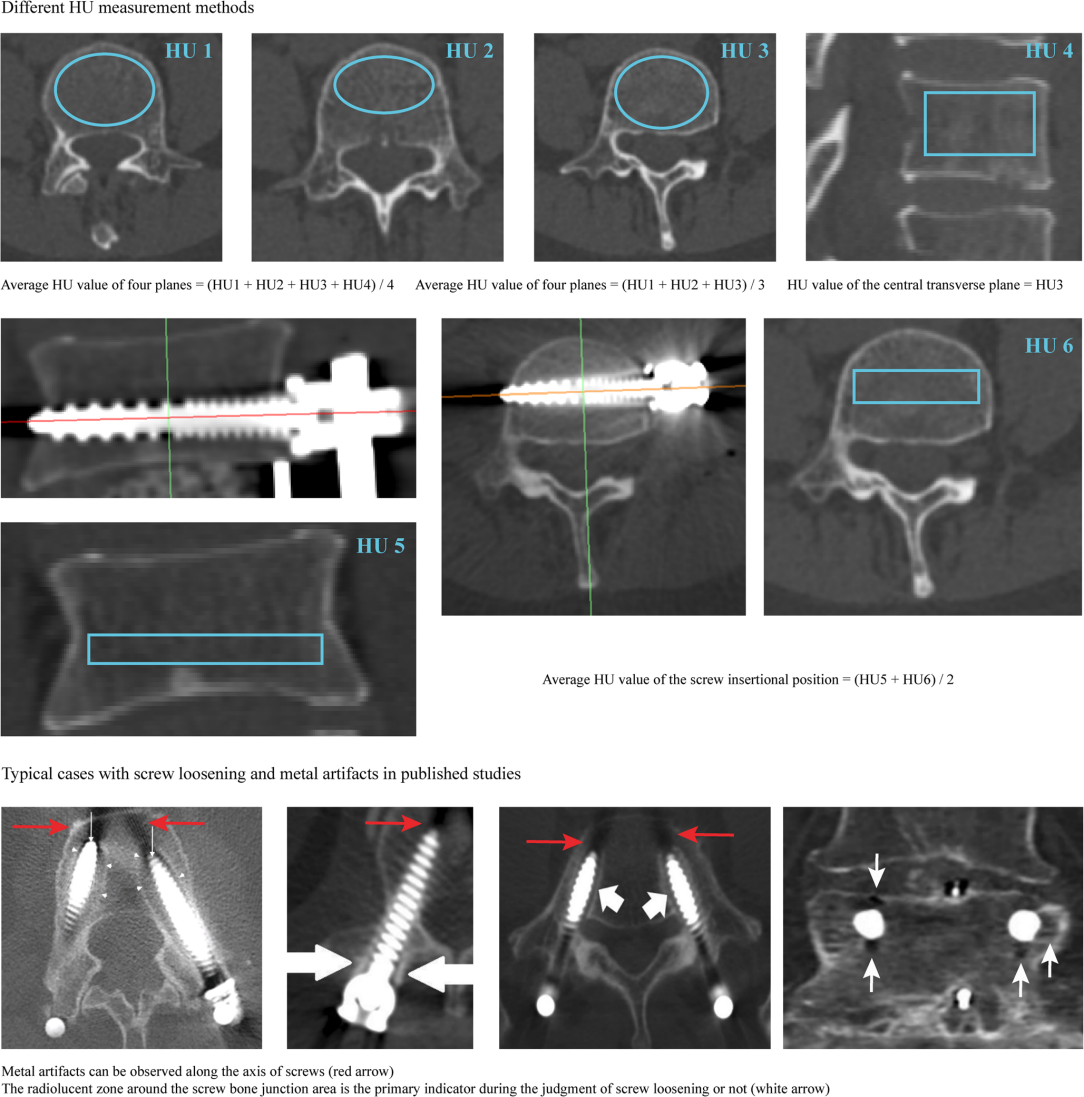
Different HU measurement methods and the judgment of screw loosening status by CT imaging data (Metal artifacts can be observed along the axis of the screw, and the radiolucent zone around the screw bone junction area is the primary indicator during the judgment of screw loosening or not) [5, 27–29]

All patients underwent lumbar CT three times in the radiology center of our hospital, including 1 week before, 1 week after, and around 1 year (within 11 to 13.5 months) after OLIF surgery. Imaging data from different CT scans play different roles in this study. Specifically, all HU value measurement procedure was performed in the preoperative CT scan, screw trajectories and corresponding insertional screw position in the preoperative CT were judged according to the instant postoperative CT scan (1 week after OLIF operation), and finally, the screw loosening status was judged based on the 1 year’s postoperative CT scan. The tube voltage was set as 120 kV, and this parameter was identical to studies on the same topic [8, 19, 25, 26]. During the measurement of HU by commonly used methods, the ROI was placed in four planes: the midsagittal plane, central transverse plane, transverse plane close to the superior plane, and inferior endplate separately [4, 17, 19]. Cancellous bone included in the ROI, cortical bone, bony endplates, posterior structure osteophytes, and posterior venous plexus were excluded [9, 16, 17]. According to previously published studies, there are three commonly used HU measurement methods: the average HU value of four planes, the average value of three planes, and the HU value of the central transverse plane. In which the average of four planes was the average value of the transverse plane inferior to the superior bony endplate, the central transverse plane, the transverse plane superior to the inferior bony endplate, and the central sagittal plane (i.e., average HU values of HU1 to HU4 in Fig. 1). The average of three planes was the average value of above-mentioned three transverse planes (i.e., average HU value of HU1 to HU3 in Fig. 1); the central transverse plane’s HU was HU2 in Fig. 1 [8, 10, 18]. When evaluating HU values in the insertional screw positions, the “insertional screw position” has been judged in the preoperative CT scan according to the screw trajectory in the instant postoperative CT scan. In this process, HU values in coronal and transverse planes’ screw insertional position were separately measured (i.e., HU5 and HU6 in Fig. 1), and the average HU value of HU5 and HU6 was recorded as “HU value of the insertional screw position.”

The measurement of these imaging data was independently performed by an experienced radiologist (the radiologist, who performed the imaging measurement in this study has been a practicing general radiologist (including the MSK radiologist) for more than 10 years (He independently produced more than 2000 reports about MSK diseases)). Screw loosening was confirmed by computational tomography (CT) during the 1-year postoperative follow-up [5, 27, 28], and vertebral bodies with ≥ 1 mm width radiolucent zones around the screw were defined as screw loosening [5, 6, 8]. Vertebral bodies were divided into two groups, the screw loosening and nonloosening groups, and the cranial and caudal vertebral bodies (i.e., L4 and L5 vertebral bodies) were evaluated separately. In other words, the clinical data in this study were divided into four groups (i.e., cranial nonloosening, cranial screw loosening, caudal nonloosening, and caudal loosening).

### Statistical analyses

To judge the interobserver and intraobserver reliability, 10 patients were randomly selected. One week after the measurement of these imaging data, imaging data of these selected patients were remeasured by the radiologist and a spine surgeon (the spine surgeon who measured the HU values in this study has 5 years of experience in imaging and biomechanics-related research and nearly 1 year of learning experience in the radiology department (in the MSK imaging measurement group) during the standardized training). The intraclass correlation efficiency (ICC) was computed to identify the repeatability of measured continuous variables (ICC ≥ 0.8 represents excellent reliability) [8, 19]. The kappa values were computed to determine the interobserver and intraobserver repeatability during the judgment of screw loosening (kappa values of 0.41 to 0.60 indicated moderate reliability; 0.61 to 0.80 indicated substantial agreement; and 0.81 to 1.00 indicated excellent or almost perfect agreement) [30–32].

Statistical analyses for cranial and caudal side screw loosening were performed separately. When comparing the difference between screw loosening status (nonloosening and loosening) in both cranial and caudal vertebral bodies. The independent samples Student’s *t*-test was used for continuous variables, and the chi-square test was used for categorical variables. We performed binary logistic regression to identify independent risk factors for screw loosening. To investigate whether demographic data affect the risk of screw loosening, patients’ sex, BMI, and age were enrolled in regression analyses. In contrast, because excellent consistency between HU values measured by different methods existed (according to the computation of ICC), HU values were included in the regression analyses separately. Univariate analyses of each potential risk factor were performed, and the variables that achieved a significance level of *p* < 0.1 were entered into multivariate analyses [33–35]. Variables with *p* < 0.05 were considered independent risk factors in the multivariate analyses. A *p* value less than 0.05 indicated a significant difference [33–35]. Finally, we performed ROC curve analyses to assess the predictive value of HU measured by different methods, and the area under the curve (AUC) was calculated as an indicator to judge the predictive performance. A *p* value less than 0.05 indicated a significant difference.

## Results

### Patient collection and screw loosening rates

A total of 56 patients (30 males and 26 females) with an average age of 56.57 ± 11.96 years treated by L4-L5 segment OLIF with ALSR screw fixation were recorded. There were no significant differences in patients’ BMI and sex in the nonloosening and screw loosening groups. The overall incidence rate of screw loosening was 35.71% (40/112), the screw loosening rate of the vertebral body on the cranial side was 42.86% (24/56) and that of the caudal vertebral body was 28.57% (16/56). Patients in the cranial screw loosening group were older than those in the nonloosening group, but there was no significant difference in age between the caudal side’s nonloosening and screw loosening groups (Table 1). The interobserver and intraobserver reliability of continuous variable measurement was excellent, with ICCs of 0.884 and 0.855, and the kappa values during the judgment of screw loosening were 0.778 and 0.759, respectively (Table 2).

**Table 1:** Demographic data for patients with and without screw loosening

	Nonloosening	Loosening	*p* value
Cranial			
Age	56.75 ± 12.7	63.58 ± 9.86	0.033*
BMI	25.2 ± 3.63	24.88 ± 3.11	0.729
Sex	20/10 (M/F)	10/14 (M/F)	0.122
Caudal			
Age	58.08 ± 12.79	63.69 ± 8.69	0.114
BMI	25.11 ± 3.45	24.94 ± 3.34	0.765
Sex	23/7 (M/F)	18/8 (M/F)	0.531

*Statistical significance in the multivariate regression analysis (p < 0.05)

**Table 2:** Validation of measured values repeatability.

	Interobserver	Intraobserver
ICCs of continuous variables	0.884	0.855
Kappa values of union status	0.788	0.759

### Identification of independent risk factors for screw loosening

When identifying the risk factor for cranial screw loosening, based on the results of univariate logistic regression analyses, patients' age and HU values measured by both methods were entered into the multivariate analysis. Considering the excellent consistency between HU measured by these methods, the multivariate analysis of different HU values was performed separately. The results showed that reducing HU, measured by these four methods, was an independent risk factor for screw loosening on the cranial side (Table 3).

**Table 3:** Logistic regression analysis of the cranial screw loosening

	OR	95% CI		*p*
Univariate analysis				
Gender	2.333	0.791	6.885	0.125
Age	1.053	1.003	1.106	0.039^#^
BMI	0.972	0.83	1.138	0.723
HU (average of four planes)	0.976	0.959	0.993	0.005**
HU (average of three planes)	0.979	0.963	0.995	0.011*
HU (central transverse plane)	0.976	0.960	0.993	0.005**
HU (screw insertional position)	0.968	0.951	0.986	0.000**
Multivariate analyses				
Age	1.038	0.984	1.095	0.172
HU (average of four planes)	0.978	0.961	0.996	0.015*
Age	1.039	0.986	1.095	0.152
HU (average of three planes)	0.981	0.965	0.998	0.032*
Age	1.042	0.988	1.1	0.129
HU (central transverse plane)	0.978	0.961	0.992	0.012*
Age	1.024	0.964	1.088	0.444
HU (screw insertional position)	0.971	0.953	0.988	0.001**

^#^Variables that achieved a significance level of *p* < 0.1 in the univariate analysis

*Statistical significance in the multivariate regression analysis (*p* < 0.05)

**Statistical significance in the multivariate regression analysis (*p* < 0.01)

Concerning the caudal side, there were no significant age differences between patients with credible fixation and screw loosening (Table 1). The *p* value of age during the univariate logistic regression analysis was higher than 0.1 (Table 4). Considering that only the *p* values of mean HU values were < 0.1 in the univariate logistic regression analysis, multivariate analysis was not performed. The reduction of vertebral bodies' HU and screw holding planes’ HU were regarded as independent risk factors for screw loosening in the caudal vertebral body (Table 4).

**Table 4:** Logistic regression analysis of the caudal screw loosening

	OR	95% CI		*p*
Univariate analysis				
Gender	1.739	0.54	5.604	0.354
Age	1.042	0.99	1.097	0.117
BMI	0.985	0.828	1.17	0.86
HU (average of four planes)	0.957	0.933	0.982	0.001**
HU (average of three planes)	0.96	0.937	0.983	0.001**
HU (central transverse plane)	0.959	0.936	0.983	0.001**
HU (screw insertional position)	0.941	0.91	0.974	0.001**

**Statistical significance in the univariate regression analysis (*p *< 0.01)

Multivariate regression analysis has not been performed when identifying the independent risk factor for the caudal screw loosening

### Parameter prediction values for screw loosening

**Fig. 2:**
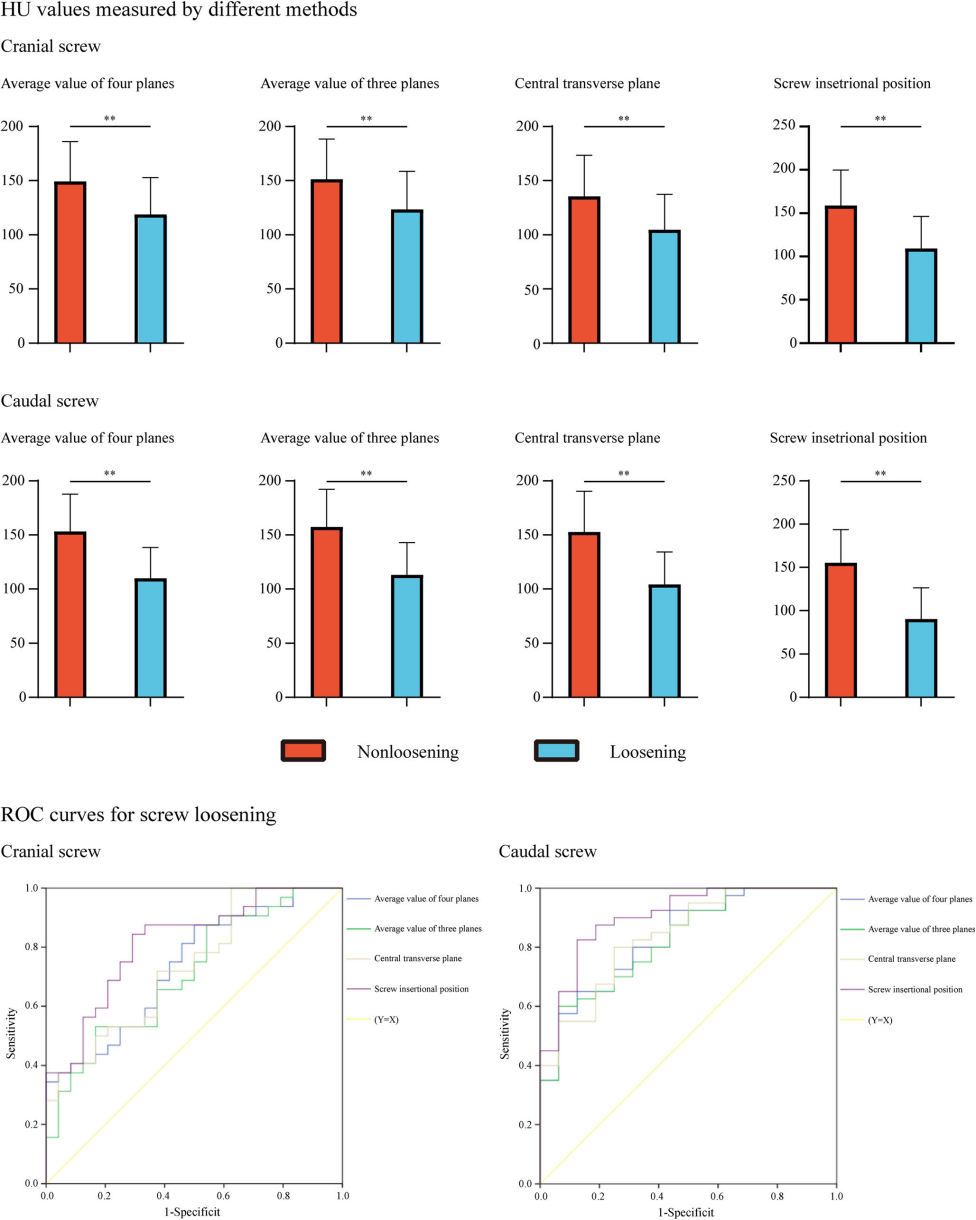
The mean HU value measured by the four methods in the loosening group and the nonloosening group and ROC curves of different HU measurement methods

The mean HU values in the nonloosening group were significantly higher than those in the screw loosening group, regardless of whether the HU was measured by the abovementioned four methods. Differences in mean HU values in nonloosening and screw loosening vertebral bodies were highest in the screw insertional positions on both cranial and caudal sides (Fig. 2). The difference in HU value in the cranial side screw insertional positions was 49.58 and that of the caudal side was 64.73.

**Fig. 3:**
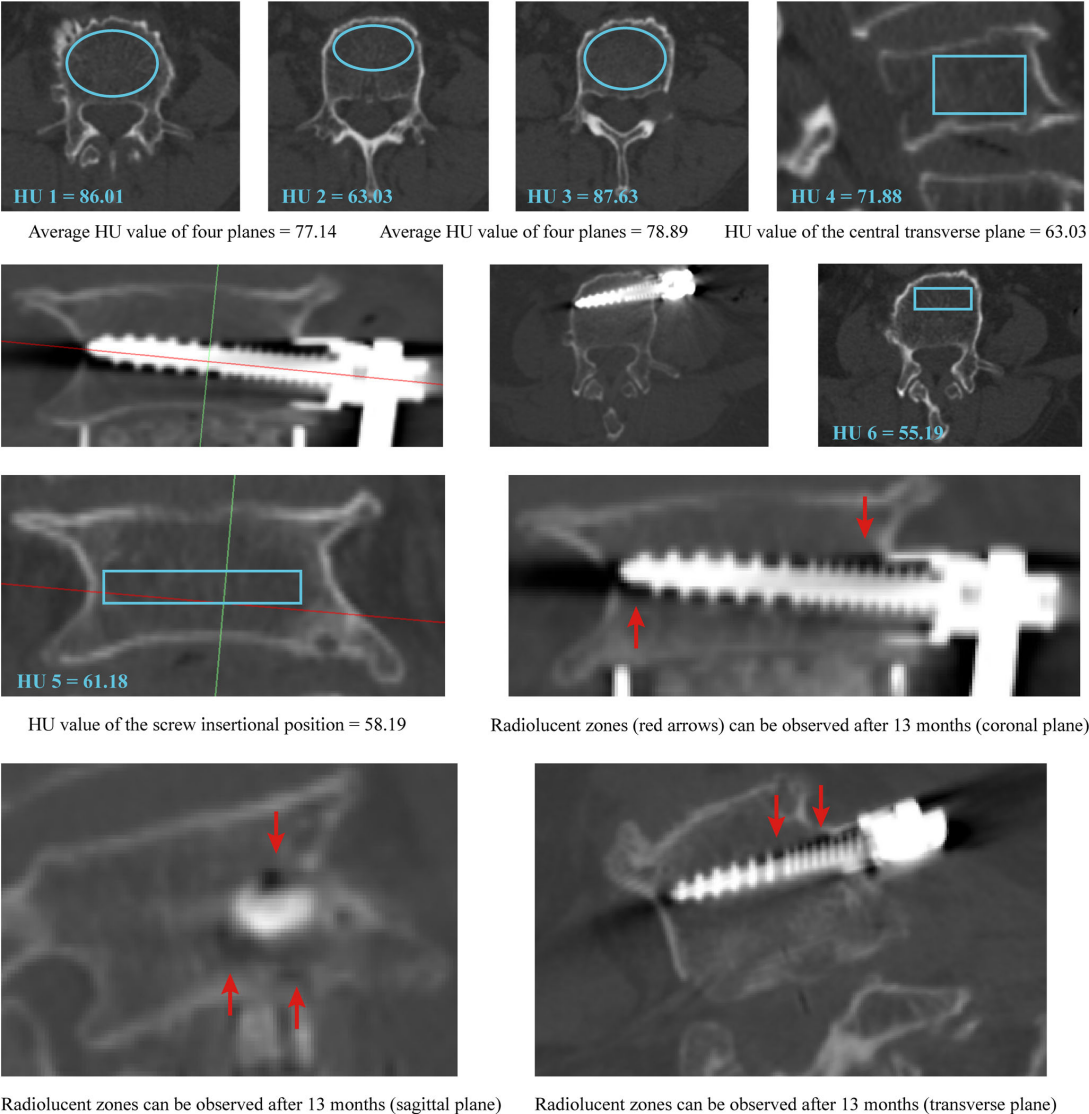
A typical case for the better predictive ability of the screw insertional position’s HU in patients with screw loosening: A 73-year-old female who suffered spinal canal stenosis and was treated by L4-L5 segment OLIF with ALSR fixation. Screw loosening of the cranial vertebral body can be observed in the CT scan at 13 months postoperatively. The HU value measured in the insertional screw position was lower than that measured by the other three methods

**Fig. 4:**
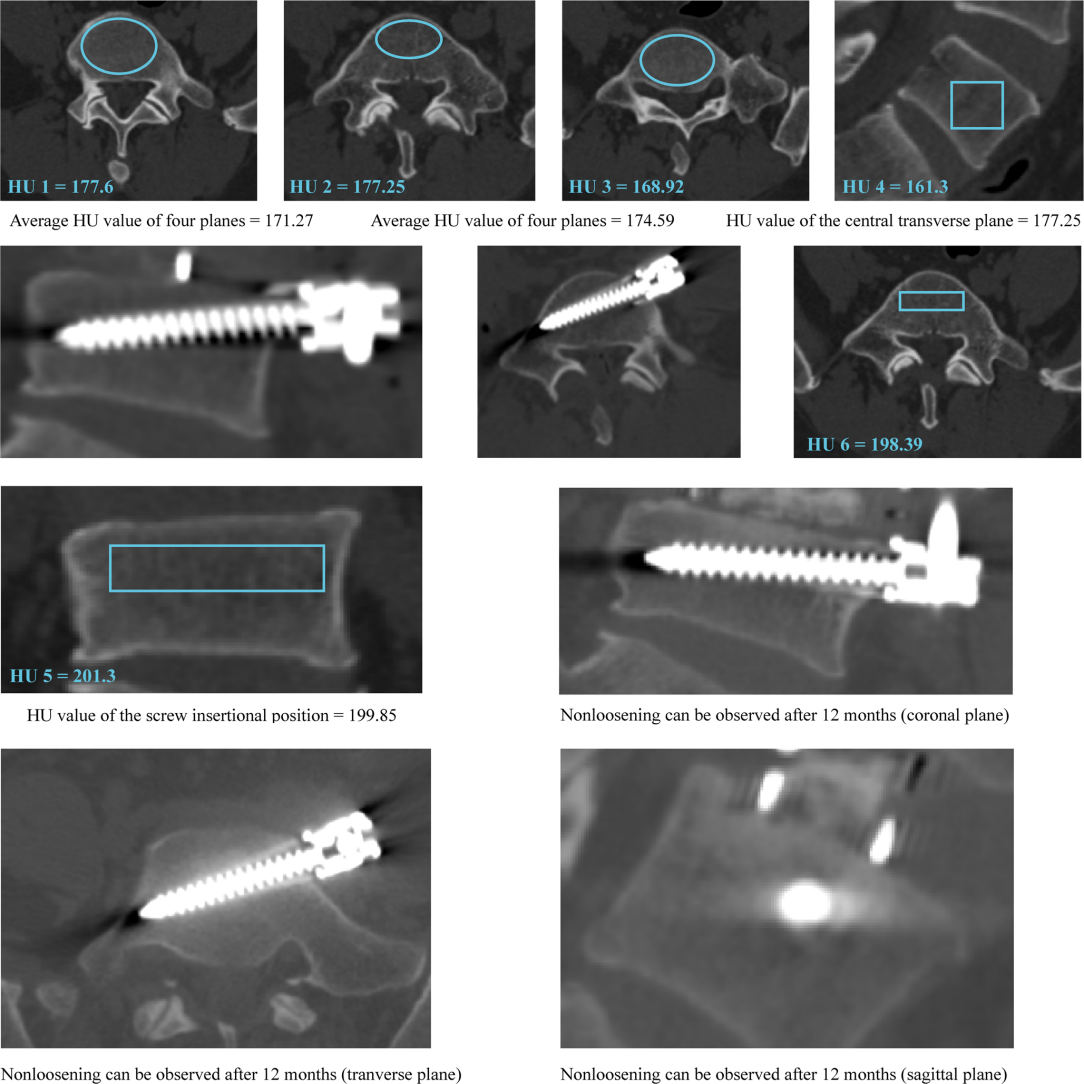
A typical case for the better predictive ability of the screw insertional position’s HU in nonloosening patients: A 48-year-old male who suffered spinal canal stenosis and was treated by L4-L5 segment OLIF with ALSR fixation. Credible screw fixation (nonloosening) can be observed in the caudal vertebral body in the CT scan at 12 months postoperatively. The HU value measured in the insertional screw position was higher than that measured by the other three methods

We performed ROC curve analyses to assess the predictive value of different HU measurement methods in the cranial and caudal vertebral bodies separately (Figs. 2, 3, and 4 and Table 5). Compared with commonly used HU measurement methods, we can compute the highest AUC values of the screw insertional position during the prediction of screw loosening in both cranial and caudal vertebral bodies. The AUC of insertional screw position was 0.816 during the prediction of cranial screw loosening and 0.905 during the prediction of caudal screw loosening.

**Table 5:** The cut-off value, sensitivity and specificity of four measurement methods for predicting screw loosening (AUC of HU values measured in the screw insertional position was higher than other methods)

	Cut-off value	Sensitivity	Specificity	AUC
Cranial vertebral body				
HU (average of four planes)	105.56	0.875	0.478	0.733
HU (average of three planes)	106.31	0.906	0.417	0.711
HU (central transverse plane)	110.37	0.719	0.583	0.736
HU (screw insertional position)	119.4	0.875	0.667	0.816
Caudal vertebral body				
HU (average of four planes)	107.3	0.928	0.562	0.831
HU (average of three planes)	126.02	0.75	0.687	0.825
HU (central transverse plane)	118.06	0.775	0.75	0.839
HU (screw insertional position)	113.75	0.875	0.812	0.905

## Discussion

In this study, to verify if HU values measured in the insertional screw position may better predict the risk of screw loosening. We compared the predictive performances (AUC values) of different HU measurement methods. The current results proved that HU values directly measured from the insertional screw position were a better predictor (highest HU value, AUC = 0.816 for the cranial and 0.905 for the caudal screw loosening) than other methods during screw loosening prediction.

The T score measured by DXA is a commonly used indicator of osteoporosis [10, 36]. The loss of integration in the cancellous-screw interface is the most important mechanism of screw loosening for patients with osteoporosis [37, 38]. Based on above-mentioned limitations, the predictive performance of DXA in BMD prediction is lower than HU values [6–8, 16, 19, 36], and given that the difference in HU values between nonloosening and loosening patients was highest in the insertional screw position group, we believe that the insertional screw position’s HU value could better represent the BMD in the screw-bone integrated area. Moreover, considering that its AUC was higher than that of the other groups, the elimination of regional BMD differences in the cancellous bone should optimize the predictive performance of HU during the evaluation of screw loosening risk.

Admittedly, the overall screw loosening rate was 35.71% (40/112), higher than that of other studies whose patients were fixed by bilateral pedicle screws (BPS) [8, 39, 40]. Differences in imaging measurement methods can explain, or at least partly explain, the higher screw loosening rate in our study. Specifically, the status of screw fixation (loosening or not) was judged by the X-ray image in other studies and determined by the CT scan in our study [4, 6, 41]. According to the published study, the sensitivity and specificity of the CT scan are better than the two-dimensional imaging examination (i.e., X-ray) [28]. In studies evaluating the risk of screw loosening in bilateral pedicle screw fixation patients, the screw loosening rate judged by CT was 38.8% [5]; this screw loosening rate was higher than that measured by X-ray (30.6%) [8]. Although there are indeed differences in patients’ surgical methods and demographic data in these studies, these studies can still partially explain the higher screw loosening rate in our study compared with Zou et al [8]. Moreover, in this study, the cutoff value of HU measured by the average three planes’ HU was 113.21, which is slightly higher than that reported by Zou et al. In their study, the cutoff value measured by the same method was 110 in patients fixed by BPS, and the status of screw loosening was judged by X-ray rather than CT scan. Considering the higher sensitivity of CT in the examination of screw loosening, approximate cutoff values of different fixation methods may indicate that the loosening risk of ALSR is not significantly higher than that of BPS.

Meanwhile, studies have also repeatedly proven that the fixation stability of BPS is better than that of ALSR screw fixation [42–44]. Moreover, our numerical simulations also computed the stress distribution of ALSR and BPS in OLIF models. The computational results show that the maximum equivalent stress of BPS was lower than that of ALSR under all loading conditions. Considering that the initial stress concentration triggers screw loosening, we believe that the relatively higher screw loosening risk can also be partly explained biomechanically. Taken together, the combination of a more sensitive imaging examination (i.e., CT) and a poor biomechanical environment could partly explain the higher screw loosening rate in this study.

Screw fixation is a feasible method to construct instant postoperative stability of the surgical segment in LIF patients, and the surgical segment's stability could provide an ideal biomechanical environment for osteogenesis during the LIF process. In other words, in patients with screw loosening, loss of bone-screw integration will lead to poor instant postoperative stability, resulting in cage migration, subsidence, and nonunion that will adversely affect patients’ prognosis and increase the rate of revision surgery [45, 46], especially for OLIF patients without direct posterior approach decompression. In other words, it is of great significance to reduce the risk of screw loosening to improve the clinical prognosis for OLIF patients.

Although studies proved that the measurement of HU values in the pedicle region could provide a good predictive performance of pedicle screw loosening [22, 24, 25, 47], this was the first study to identify the ideal predictive performance of ALSR insertional position HU values. We believe this study was of great significance to optimize OLIF patient prognoses. Specifically, detailed preoperative planning is an essential guarantee for surgical efficacy. A lumbar CT scan is a standard preoperative examination for LDD patients, and HU can be measured easily without extra expense and radiation. More significantly, the trajectory (the insertional screw position and angles in different sections) of the ALSR screw is highly adjustable [44, 48, 49]. By measuring HU values in different planes of the coronal and transverse sections in the preoperative CT scan, the trajectory of the ALSR screw can be optimized to positions with higher HU, which should reduce the risk of screw loosening and related complications.

Indeed, this study has several limitations. First, this is a retrospective study; the conclusion of this study should be re-estimated by prospective clinical follow-up. In addition, although most of the same studies have set the follow-up period at 1 year, a longer follow-up is still necessary to further validate our findings. Additionally, the indications of patients included in this study were limited to degenerative spinal stenosis and slight degenerative spondylolisthesis, and patients with posterior decompression were also excluded from this study. Finally, the confounding effect of metal artifacts cannot be eliminated in current patients. Therefore, the conclusions in this study should be reverified in future studies on patients with different diagnoses and different surgical procedures. In future studies, an artifact-removed CT scan should be performed to reverify the current conclusion.

## Conclusions

Compared with frequently used HU measurement methods, the insertional screw position’s HU value could better represent the BMD in the screw-bone integrated area. The elimination of regional BMD differences in the cancellous area by using this method could optimize the predictive performance of HU during the evaluation of screw loosening risk. In addition, preoperative optimization of the ALSR screw trajectory (selecting the insertional screw position with higher HU) should reduce the risk of screw loosening and related complications.

## Abbreviations

ALSR: Anterior lateral single rod
BPS: Bilateral pedicle screws
LDD: Lumbar degenerative diseases
OLIF: Oblique lumbar interbody fusion surgery

## Declarations

All data collection and analysis conducted in this study were in accordance with the ethical standards of the institutional and/or national research committee and with the 1964 Helsinki Declaration and its later amendments or comparable ethical standards. For this type of study, formal consent is not required.

### Guarantor

The scientific guarantor of this publication is Prof. Jiancheng Zeng.

### Statistics and biometry

Dr. Zhuang Zhang has significant statistical expertise.

### Methodology

• A retrospective study of prospectively collected radiographic data
